# Mitochondria Localized MicroRNAs and Alzheimer’s disease

**DOI:** 10.1002/alz.086516

**Published:** 2025-01-03

**Authors:** Subodh kumar

**Affiliations:** ^1^ Texas Tech University Health and Sciences Center El Paso, El Paso, TX USA

## Abstract

**Background:**

Mitochondria plays a crucial role at synapses in providing synaptic energy, healthy synaptic function, and cognitive functions. Amyloid‐beta and phosphorylated tau protein oligomers cause severe mitochondrial defects in Alzheimer’s disease (AD), which leads to the lack of synaptic energy and impaired synapse functions in AD. MicroRNAs (miRNAs) present within the mitochondria are involved in multiple mitochondrial activities and mitochondrial function. Mitochondrial dysfunction is well established in AD; but status of mitochondria localized miRNAs are unknown in AD. This current study is focused on the identification of mitochondria localized miRNAs in AD and to unveil their possible roles in disease pathogenesis.

**Method:**

Mitochondria and cytosolic fraction were extracted from postmortem AD brains (n = 5) and cognitively normal postmortem brains (n = 5). Mitochondria purity was characterized by transmission electron microscopy (TEM) and immunoblot analysis of mitochondrial marker proteins. Mitochondria activity was determined by mitochondrial function assay. Further, total RNA was extracted from mitochondria and cytosolic fraction and subjected to miRNAs HiSeq analysis. The significantly deregulated mitochondrial miRNAs were further validated on large number of AD postmortem brain and subjected to in‐silico bioinformatic analysis.

**Result:**

TEM analysis, immunoblotting and mitochondrial function assay confirmed the extraction of intact and active mitochondria from AD and control postmortem brains. MiRNAs HiSeq analysis showed the mitochondria localized distribution of some novel miRNAs in AD and control samples. We found some miRNAs localization and differential expression in mitochondrial fraction relative to cytosolic fraction. Our validation analysis unveiled previously unknown, most potential mitochondria localized miRNAs in AD. Further, *in silico* bioinformatic analysis revealed the critical roles of mitochondrial localized miRNAs in several mitochondrial function and synaptic pathways in AD.

**Conclusion:**

Our study discovered some novel mitochondria localized miRNAs, which could be a potential therapeutic target to retrieve mitochondrial and synaptic dysfunction in AD.

